# Beyond Immunosuppression: The Multifaceted Functions of Tumor-Promoting Myeloid Cells in Breast Cancers

**DOI:** 10.3389/fimmu.2022.838040

**Published:** 2022-03-03

**Authors:** Céline Blaye, Thomas Boyer, Florent Peyraud, Charlotte Domblides, Nicolas Larmonier

**Affiliations:** ^1^Centre National de la Recherche Scientific (CNRS) Unité Mixte de Recherche (UMR) 5164, ImmunoConcEpT, Bordeaux, France; ^2^Department of Medical Oncology, Institut Bergonié, Bordeaux, France; ^3^Service d’Oncologie Médicale, Centre Hospitalo-Universitaire (CHU) Bordeaux, Bordeaux, France; ^4^Department of Biological and Medical Sciences, University of Bordeaux, Bordeaux, France

**Keywords:** breast cancer, tumor-promoting myeloid cells, immunosuppression, immunotherapies, tumor microenvironment

## Abstract

Breast cancers are commonly associated with an immunosuppressive microenvironment responsible for tumor escape from anti-cancer immunity. Cells of the myeloid lineage account for a major part of this tumor-promoting landscape. These myeloid cells are composed of heterogeneous subsets at different stages of differentiation and have traditionally been described by their cardinal ability to suppress innate and adaptive anticancer immunity. However, evidence has accumulated that, beyond their immunosuppressive properties, breast cancer-induced myeloid cells are also equipped with a broad array of “non-immunological” tumor-promoting functions. They therefore represent major impediments for anticancer therapies, particularly for immune-based interventions. We herein analyze and discuss current literature related to the versatile properties of the different myeloid cell subsets engaged in breast cancer development. We critically assess persisting difficulties and challenges in unequivocally discriminate dedicated subsets, which has so far prevented both the selective targeting of these immunosuppressive cells and their use as potential biomarkers. In this context, we propose the concept of IMCGL, “pro-tumoral immunosuppressive myeloid cells of the granulocytic lineage”, to more accurately reflect the contentious nature and origin of granulocytic cells in the breast tumor microenvironment. Future research prospects related to the role of this myeloid landscape in breast cancer are further considered.

## Highlights

Beyond their cardinal immunosuppressive properties, many subsets of myeloid cells are equipped with multiple tumor-promoting functions impacting most steps of cancer development.

## Introduction

Centered for years on the intrinsic characteristics of tumor cells, the field of cancer research has evolved toward the notion that cancers emerge and develop in a dedicated tumor-promoting environment. The cross-talks between malignant cells and components of this tumor-specific landscape dictate the fate of cancer (persistence or elimination) and further shape the nature of this microenvironment (1, 2). In this context, the influence of the immune system on cancer development has been widely evidenced, and many strategies have been developed to induce, restore and enhance anti-cancer immunity. Successes of these immune-based approaches in inducing efficient anti-tumor responses and improving cancer patient survival have brought some of them to the forefront of cancer therapeutics in recent years (3). However, it has also become clear that cancers can escape from immune detection and destruction by many mechanisms resulting in the establishment of an immunosuppressive tumor environment, which represents a major obstacle for efficient immunotherapies. Compelling evidences have indicated that inhibition of these immunosuppressive networks is an important prerequisite to uncover the full potential of immune-based interventions. It is noteworthy that, although several immunotherapies provide clinical benefits in melanoma, lung, bladder and colon cancers, breast cancer patients have yet to fully experience these breakthroughs. Indeed, except for triple-negative cancers which are more immunogenic and have obtained FDA approval of immunotherapies in the neo-adjuvant (4) and metastatic (5) settings, most immune-based therapeutic attempts in breast cancers have ended in failure.

For many years, the environment of breast tumors has been described as “immunologically cold”, as defined by the sparsity or absence of tumor-infiltrating lymphocytes (TILs) (6). This description is somewhat inaccurate insofar as it usually does not take into account cells of myeloid origin, despite their many diverse roles in the environment of mammary cancers. The lack of anti-tumoral immune response in breast cancers has indeed been associated with a hostile immuno-inhibitory microenvironment, the major components of which being cells of myeloid origin (7). Tumor-associated macrophages (TAMs), tumor-associated neutrophils (TANs), tolerogenic dendritic cells (tDC) and immature subsets of myeloid cells endowed with immunosuppressive properties termed “myeloid-derived suppressor cells” (MDSCs) have been identified as such myeloid subpopulations, present not only within the tumor environment, but also at the sites of priming of antitumoral immune responses (secondary lymphoid organs), in the bloodstream and in the pre-metastatic and metastatic sites.

**Figure 1:**
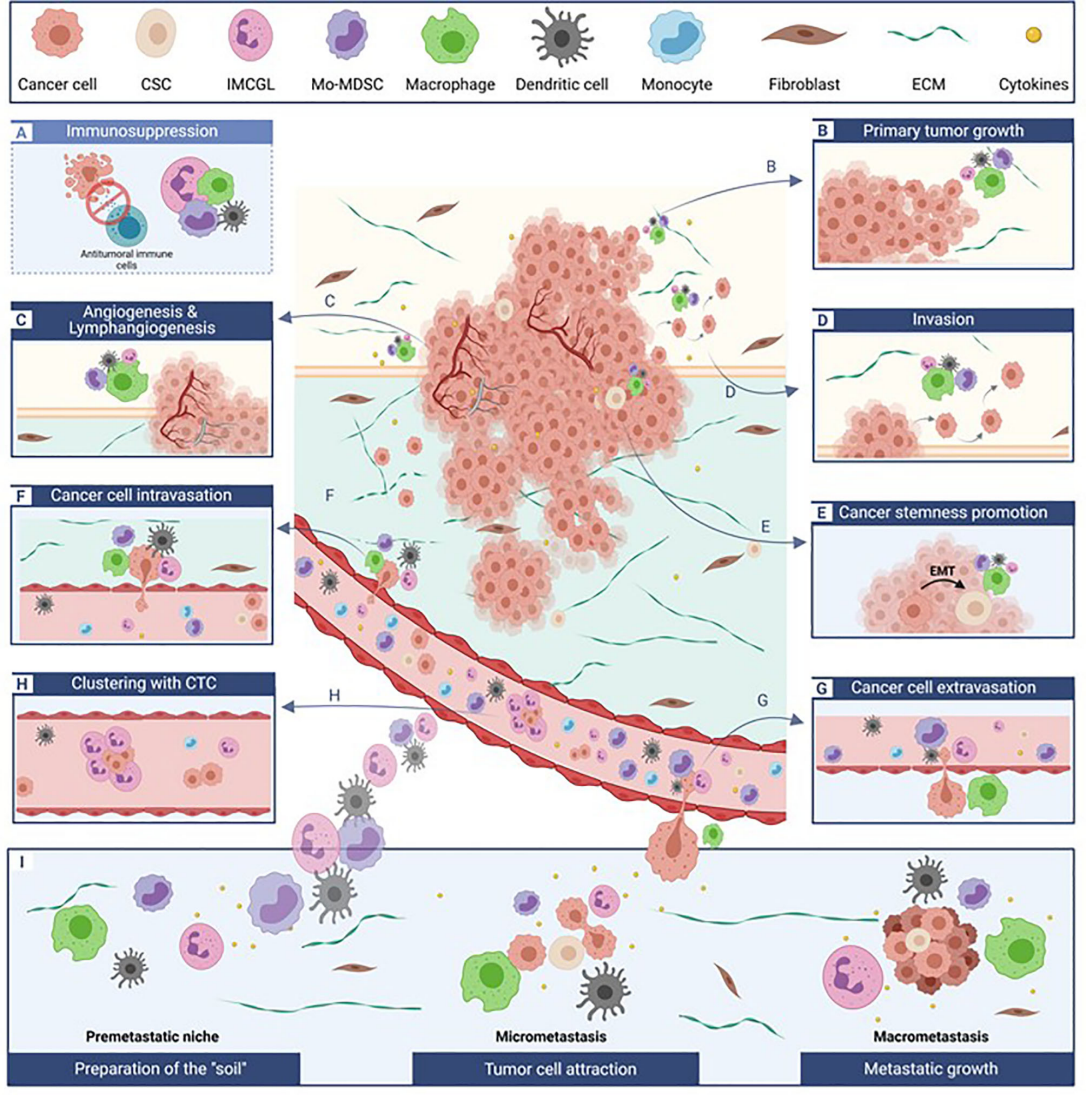
Tumor-promoting myeloid cells critically affect multiple and distinct steps of cancer development. Besides impairing anti-tumor immunity. **(A)**, dedicated subpopulations of myeloid cells differentially impact primary tumor survival and growth **(B)**, tumor vascularization **(C)**, local tissue invasion **(D)** cancer stemness **(E)**, tumor cell intra- **(F)** and extravasation **(G)** in and from blood vessels, associate with circulation tumor as beneficial clusters **(H)**, and participate to metastatic site preparation and development **(I)**. The relative contribution of each myeloid cell subset to a specific process (each illustrated in a separate box) is depicted by the differential size of the cells. CSC, cancer stem cells; CTC, circulating tumor cells; ECM, extra-cellular matrix; EMT, epithelial-to-mesenchymal transition; IMCGL, immunosuppressive myeloid cells from granulocytic lineage; Mo-MDSCs, monocyctic myeloid-derived suppressor cells.

Besides their ability to impair anti-tumor immunity at different steps of immune responses (initiation, priming, effector stages), these myeloid cells are also endowed with a large array of “non-immunologic” tumor-promoting functions. They can indeed contribute to the epithelial-to-mesenchymal transition (EMT), participate to local tissue invasion at the primary tumor site, foster blood or lymphatic vessel intravasation and extravasation of migrating cancer cells, associate with circulating tumor cells protecting them in the bloodstream, and prepare the pre-metastatic niches thus enhancing metastatic processes. Furthermore, these myeloid cells can also directly promote primary tumor cell survival and proliferation and foster tumor neoangiogenesis and cancer cell stemness (**Figure 1**). The role of breast cancer-induced myeloid cells in resistance to chemotherapy as well as endocrine therapy has also been described, making them potential targets for the development of new immunotherapies.

Recently, major advances in the characterization of the phenotypes, functions and origins of myeloid cell subpopulations in breast cancers have been made, particularly by single cell RNA sequencing approaches. In this review, we discuss the equivocal identify of some subsets, particularly “polymorphonuclear-MDSCs” (“PMN-MDSCs”) and “immunosuppressive neutrophils”, and examine and discuss the polyvalent tumor-promoting functions of these myeloid cells in the breast cancer environment in light of recent literature, with a specific emphasis on the “non-immunologic” pro-tumoral properties of these multitasking cells.

## Macrophages in Breast Cancers: A Multifunctional Impact on Tumor Promotion

### Macrophage Phenotype and Function in the Context of Breast Cancer

Macrophages have been one of the most widely studied population of myeloid cells in cancer, specifically in the context of breast malignancies. In breast cancer patients, tumor-associated macrophages (TAM) have been associated with aggressive features (size ≥ 2 cm, higher tumor grade, higher Ki67) and estrogen receptor (ER) negative breast cancers (8). However, the prognostic value of these cells remains controversial and depends on the cancer subtype, the macrophage subset (M1 *vs* M2, see below) and their localization (9). Indeed, some authors have described an improved survival of ER^-^ or triple-negative breast cancer (TNBC) patients with a CD163^+^CD68^+^ macrophage infiltrate (10), while others correlated the presence of tumor-infiltrating CD163^+^ macrophages with a worse prognosis of TNBC patients (11). In a gene-expression based study using a CIBERSORT deconvolution method, macrophages have been associated with a significant poorer outcome in both ER^+^ and ER^-^ BC patients, and were predictive of a worse response to chemotherapy in ER^-^ patients (12). Independently of BC subtype, immune population clustering identified 2 clusters enriched in pro-tumorigenic macrophages, which have been associated with significantly worse outcome in BC patients (12).

The polyvalent functions and the high degree of plasticity of macrophages are partly responsible for these conflicting results. Macrophages have originally been broadly discriminated in two different types with opposite roles. “M1” macrophages have been described as classically activated, pro-inflammatory, anti-tumoral effectors, whereas “M2” macrophages correspond to alternatively activated cells endowed with “wound-healing” and tumor-promoting functions. M1 and M2 represent in fact two extreme polarization states of a highly plastic differentiation program controlled by environmental cues (13). M1 macrophages can be induced by TLRs ligands such as LPS, and/or IFNγ. Their differentiation is driven by STAT1, IRF5, NF-kB (14). M2 macrophages are primarily induced by IL-4 and/or IL-13 (+/- IL-10) *via* the IL-4Rα receptor and their differentiation depend mainly on STAT3, STAT6, IRF4 activation. Different markers have been used to distinguish between M1 and M2 macrophages, but most of these molecules are expressed by both types, although at different levels (14). In immunohistochemical studies, CD68 is often used as a “pan-macrophages” marker. M1 are described as iNOS-expressing cells, with high expression of MHC class II and detectable co-stimulatory molecules CD80 and CD86. M2 macrophages conventional markers include CD163, the scavenger receptor CD204 and mannose receptors CD206, as well as a high expression of Arg1 (9). However, this M1/M2 dichotomy has been challenged and it may not be fully relevant in the context of chronic, non resolutive inflammation such as cancer (14). In fact, recent transcriptomic data, RNA sequencing and mass-cytometry analyses argue for a more complex and heterogeneous phenotypic identity of breast cancer-associated macrophages. A single-cell RNA-seq analysis of 8 tumors (matched to healthy tissues from the same patients) uncovered numerous clusters of immune cells. Among them, three different clusters of TAMs were described (15). Whether TAM may originate from bone-marrow-derived monocytes or from tissue-resident macrophages, which derive from embryonic macrophages that colonize developing organs during the process of embryogenesis and that persist in mature developed adult organs, has been debated. The abovementioned study indicated that the three identified distinct TAM clusters originate either from monocytes or from resident macrophages. Interestingly, among these TAMs, the M1 gene signature correlated with that of M2, advocating for a simultaneous activation of these different genes (15). Along these lines, a mass-cytometry analysis of 144 breast tumors (compared with 46 matched juxta-tumoral tissue and four mammoplasties from cancer-free individuals) defined 19 clusters of myeloid cells and highlighted a frequent co-expression (although at different levels) of phenotypic markers of both M1 and M2 by TAMs such as CD169, CD86, CD204, CD206 and CD163 (16). Consistent with these studies, a RNA-seq analysis indicated that TAMs from breast and endometrial cancers did not exhibit typical M2 gene signatures (17). In this study, TAMs from these two different types of cancers revealed very small similarities, emphasizing the crucial role of the TME in differentially shaping macrophage phenotype and function (17). These data thus indicate that TAMs in breast cancers exhibit complex overlapping phenotypic and functional characteristics and cannot be simplistically categorized as conventional M1 *vs* M2. With regard to the origin of TAMs in breast cancers, the aforementioned single-cell RNA-seq analysis indicated that these cells can originate either from resident macrophages or from monocyte differentiation (15).

### TAMs at the Primary Tumor Site

At the primary tumor site, crosstalks between macrophages and cancer cells contribute to the recruitment and activation of TAMs, which in turn foster tumor progression through many mechanisms. Particularly, the immunosuppressive activity of these cells has been extensively described. Indeed, macrophages can suppress anti-tumoral T lymphocytes responses *via* their catabolism of L-arginine and/or tryptophan (expression of iNOS, IDO, arginase), production of immunosuppressive cytokines such as IL-10, IL-4, IL-17, CXCL1, or the expression of ligands for immune checkpoint inhibitory receptors such as PD-L1. They also produce chemoattractant chemokines that further recruit immunosuppressive cells such as neutrophils, immature DCs and/or Tregs [reviewed in (18)]. Along these lines, IL-1ß production by TAM has been shown to participate to the recruitment of immunosuppressive cells and thus to overall suppression of adaptive immune responses (19, 20). In mice, specific targeting of these immunosuppressive macrophages or inhibition of their immunoinhibitory functions can restore anti-tumor immune responses (21).

However, tumor-associated macrophages can also display pro-angiogenic functions and can promote cancer cell stemness. For instance, the transcription factor POU class 1 homeobox 1 (POU1F1, also known as Pit-1), a protein expressed by breast cancer cells, has been reported to increase macrophage recruitment and to promote their polarization towards VEGFA-expressing tumor-promoting macrophages. In turn, these macrophages foster tumor growth, angiogenesis and extravasation of breast cancer cells in a CXCL12-dependent manner *in vitro* (22). Likewise, the expression by breast cancer cells of the ID4 protein (a member of inhibitors of differentiation family of proteins), which is associated with a basal, stem-like phenotype and poor prognosis in TNBC, induces the activation of a pro-angiogenic program in macrophages with upregulation of angiogenesis-related transcripts (23). It is noteworthy that pro-tumorigenic TAM infiltration is more prominent in inflammatory breast cancer (IBC), a disease with a very poor prognosis, compared to other breast cancer subtypes. These macrophages are recruited and polarized into a pro-tumoral phenotype (upregulation of CD206, CD163 and CD209) by CSF-1, CXCL2, VEGFA and CCL18 produced by cancer cells (24). In hypoxic zones, breast cancer cells produce Oncostatin M (OSM) that induces macrophage polarization toward a tumor-promoting phenotype (higher expression of CD163, CD206, Arg1 and Cox-2) (25). Hypoxia enhances TAM expression of galectin-3, a ß-galactoside binding protein modulating TAM apoptosis, migratory and adhesive properties. These macrophages have been shown to promote the proliferation, invasion and migration of MDA-MB-231 breast cancer cells and angiogenesis *in vitro*. Furthermore, *in vivo* experiments in Balb/c mice exposed to hypoxia indicate that targeting Galectin-3 decreases lung metastasis burden and reduces endothelial cell in the primary tumor (26). The presence of sexual steroids in the TME, particularly the presence of estrogens, is a specificity of breast malignancies as breasts are made of adipose tissues producing sexual steroids in the environment. ER+ breast cancers arising in this environment are uniquely capable of responding to these signals and grow. Interestingly, it has been reported that in BC patients, estrogens induce the production of CCL2 and CCL5 within the tumor beds, leading to the recruitment and polarization of macrophages towards a pro-tumorigenic phenotype (27). The reversal of estrogen effects using Tamoxifen led to a reduced infiltration of these pro-tumoral macrophages in the primary tumor, a finding further confirmed in murine models (28).

Macrophages recruited and accumulating at the tumor site also contribute to tumor development through the promotion of cancer cell stemness *via* secretion of IL-8 and CXCL1, 2 and 3 (24). Cancer stem cells (CSCs) are described as “tumor-initiating cells” with the capability of self-renewal and asymmetric proliferation, and are characterized by a reduced sensitivity to drugs and irradiation compared to non-CSCs. These CSCs are critical for cancer dissemination and metastasis (29). The acquisition of stemness properties by cancer cells has been associated with the induction of the EMT (epithelial-to-mesenchymal transition) program. EMT is controlled by transcription factors such as *TWIST, ZEB1, SNAIL, or SLUG*, and is characterized by specific phenotypic changes whereby epithelial cancer cells acquire a mesenchymal-like phenotype, which increases their invasive and migratory potency (29). CCL2 and CXCL12 produced by breast cancer-associated fibroblasts and tumor cells promote the recruitment and differentiation of monocytes into immunosuppressive TAMs. In turn, these TAMs upregulate the expression of Vimentin, decrease the expression of E-cadherin, and induce *Twists*, *Snail* and *Slug* expression by breast cancer cells, thereby promoting the acquisition of mesenchymal and stemness properties by the latter (30). In a xenograft mouse model, CD68^+^ TAMs have been demonstrated to promote breast cancer cell stemness through expression of the transmembrane protein LSECtin, which engaged BTN3A3 (B7 family member) on breast tumor cells (31). In the same study, the authors have found a co-localization between LSECtin-expressing macrophages and breast cancer cells expressing CD90 – a stemness marker in breast cancer (31). The role of CD90 in the anchorage of monocytes/macrophages to cancer cells had previously been highlighted in a previous study (32). This CD90-dependent bound leads to the production of cytokines such as IL-6, IL-8, GM-CSF by cancer stem cells which further support cancer stemness (32). Finally, in the inflammatory context of obesity, mammary adipose tissue macrophages can be reprogrammed into a pro-inflammatory metabolically activated phenotype (MMe), which can promote tumor initiation and triple negative breast cancer stem-like properties through an IL-6/GP130-dependent mechanism (33).

### TAMs in the Metastatic Process

TAMs also play an essential role at most steps of breast cancer metastasis. As outlined above, these myeloid cells contribute to breast cancer EMT and stemness, two essential initial steps required for tumor systemic dissemination (24, 30–32). TAMs located in the tumor beds or at their vicinity have also been reported to promote intravasation of migrating cancer cells from the primary tumor in blood vessels, while TAMs at the metastatic sites may contribute to the preparation of the pre-metastatic niches before colonization by cancer cells, and enhance breast cancer cell extravasation from blood capillaries in distal metastatic tissues (9).

Many reports have described the influence of TAMs in breast cancer cells intravasation, but the underlying mechanisms *in vivo* have not been extensively studied. A real-time imaging analysis in the MMTV-PyMT mouse model has indicated that VEGF-A produced by Tie2-expressing macrophages induced the loss of vascular junctions and transient vascular permeability, allowing for breast tumor cell intravasation (34). More recent studies have identified proteins involved in pro-tumoral macrophage promotion of cancer cell invasion *in vitro* assays. Chitinase 3-like protein 1 (CHI3L1, a glycoprotein highly expressed in solid tumors) secreted by macrophages has been shown to enhance breast cancer cell invasion, migration and adhesion. CHI3L1 has been detected in the sera of patients with breast carcinomas but not in healthy individuals. Analysis of GEO databases has indicated that CHI3L1 is associated with a worse prognosis in breast cancer patients (35). Use of 2D, 3D and Transwell migration assays have also underlined the role of pro-tumorigenic macrophage-secreted CCL-18 in promoting breast cancer cell migration (36). TAMs can also indirectly foster tumor dissemination by promoting the expansion of pro-metastatic neutrophils by an IL-1ß-dependent mechanism (see neutrophil section below) (37). Even in early and non-invasive breast cancers (*in situ* carcinomas) in mice, CCL2-recruited tumor-infiltrating macrophages with pro-tumorigenic features (CD206^+^/Tie2^+^), downregulate expression of E-cadherin by malignant cells, thus destabilizing cell-cell junctions, which leads to cancer dissemination and metastasis. These data advocate further for a decisive role of these cells in the establishment of metastatic disease (38).

Macrophages can also contribute to the preparation of the pre-metastatic niches and the promotion of breast cancer cell extravasation from blood vessels in distant sites. Indeed, monocytes, recruited to pre-metastatic niches by the CCL2, have been reported to quickly differentiate into pro-metastatic macrophages, which contribute to metastatic disease (9, 18). More recently, the presence of CYP4A-expressing TAMs in uninvolved tumor draining lymph nodes has significantly been correlated with the expression of markers associated with pre-metastatic niche formation (VEGFR1, S100A8 and fibronectin), and with a reduced overall and relapse-free survival of patients. In the same study, the specific targeting of CYP4A using pharmacological approaches in 4T1 breast tumor-bearing mice reprogrammed tumor-infiltrated TAMs with a F4/80^+^CD206^+^ phenotype into TAM with a F4/80^+^iNOS^+^ “anti-tumor” phenotype, and reduced lung metastatic burden by impairing the preparation of the pre-metastatic niches (39). At the metastatic sites, macrophages further promote metastatic disease development by fostering vessel formation and directly enhancing cancer cell growth and survival, through the expression of VEGFA and downstream upregulation of MMP-9 (40).

TAMs have been recently described to promote lymphangiogenesis by two different mechanisms. First, expression of podoplanin, a transmembrane glycoprotein implicated in cell motility and adhesion, has been detected on TAMs at the vicinity of lymphatic vessels in the breast TME. The binding of podoplanin to the galectin 8 protein, a secreted glycan-binding protein expressed by lymphatic endothelial cells, promotes the secretion of the pro-migratory integrin ß1 by macrophages, which in turn fosters their migration and binding to lymphatics vessels where they induce matrix remodeling and promote vessel growth and lymphoinvasion. In the same study, podoplanin-expressing TAMs were associated with lymph node invasion and organ metastasis in a small cohort of breast cancer patients (41). Second, signaling through the sphingolipid sphingosine-1-phosphate receptor 1 (S1PR1) expressed on TAMs induced macrophage NLRP3 inflammasome expression, leading to the production of IL-1ß, which in turn directly acted on lymphatic endothelial cells to promote lymphangiogenesis. In mice deficient in S1PR1 in macrophages, lymphangiogenesis and metastatic growth are impaired. In human, NLRP3 expression in macrophages correlated with lymph nodes invasion and distant metastasis (42). Consistent with the aforementioned observations, *in vitro* experiments confirmed the role of macrophage-derived IL-1ß in the promotion of breast cancer cell adhesion to human lymphatic endothelial cells (43).

## Breast Cancer Promotion by “Myeloid-Derived Suppressor Cells (MDSCs)”: beyond the Suppression of Anti-Tumor Immunity

### “MDSCs”: A Functional Definition Rather Than a True Biological Subtype

The term “myeloid-derived suppressor cells (MDSCs)” was initially proposed by Gabrilovich et al. in 2007 in an effort to globally describe a heterogeneous population of myeloid cells exhibiting an immature phenotype and endowed with immunosuppressive functions (ability to suppress T lymphocytes), which accumulate in large numbers in the context of cancer (44). These cells have drawn intense scrutiny over the last 20 years and a considerable amount of data has been provided related to their participation to the complex immunoregulatory networks responsible for tumor immune escape. It has also become clear that they contribute to tumor development and dissemination through many different “immune-unrelated” mechanisms. “MDSCs”, in the context of cancer, derive from bone marrow hematopoietic precursors through aberrant myelopoiesis induced by tumor-derived factors (45). Many chemokines have been involved in MDSCs generation and recruitment to primary tumor sites or pre-metastatic niches, such as CXCL1, CXCL2, CXCL5, CXCL12, GM-CSF, G-CSF, M-CSF, VEGF, IL-6, IL-1ß or ß-FGF (46). More recently, breast cancer cells-derived exosomes have been shown to induce “MDSCs” from bone marrow myeloid progenitors (47), or to lead to their recruitment (48). In the specific estrogen-rich environment of breast cancer, these hormones have been shown to induce “MDSCs” recruitment *via* the activation of cancer associated fibroblasts, which in turn secrete CXCL12 (49). “MDSCs” have been defined as myeloid cells blocked at different stages in their differentiation toward mature terminally differentiated subsets such as macrophages and are thus associated with different degree of immaturity. This hallmark is however not always explored in many studies on “MDSCs”. The immunosuppressive capabilities of these cells, enabling them to block innate and adaptive anti-tumoral immune responses, represent their primary characteristic which must be systematically investigated for their identification as such (50).

If the term “MDSCs” was designed to globally encompass immature myeloid cells with many common features, it has nonetheless become confusing, particularly because it has contributed to consider these cells as a unique population of myeloid cells. However, “MDSCs” are made of highly heterogeneous populations, including cells from the monocytic and granulocytic lineage. In human, monocytic (M)-MDSCs have been defined as Lin^-^CD33^+^CD11b^+^HLA-DR^low/-^CD14^+^CD15^-^, granulocytic (G) or polymorphonuclear (PMN)-MDSCs as CD33^+^CD11b^+^HLA-DR^low/-^CD14^-^CD15^+^CD66b^+^, and “early stage” (more immature MDSC) (eMDSCs) as CD33^+^HLA-DR^-^Lin^-^ (Lin: CD3, CD19, CD20, CD56, CD14, CD15) (50). In mice, MDSCs are CD11b^+^/Gr1^+^ cells, with Gr1 composed of two molecules, Ly6C (expressed on monocytic cells/M-MDSCs), and Ly6G (expressed on granulocytic cells/PMN-MDSCs) (51). As a main pitfall in the field, in many preclinical studies the phenotypical characterization of “MDSCs” has been limited to CD11b^+^Gr1^+^, which does not allow to discriminate between monocytic and granulocytic myeloid cells, each subset being endowed with distinct functions (52).

Granulocytic MDSC or “PMN-MDSC” constitute the majority of the MDSC pool in many cancers. However, phenotypically and functionally, these “PMN-MDSC” can hardly be distinguished from pro-tumoral immunosuppressive neutrophils and share the same phenotype as differentiated granulocytes. For these reasons, and since this overlap between PMN-MDSC and tumor-associated neutrophils remains a significant challenge in the field, we will discuss their phenotype and function together with that of cells of the granulocytic lineage in a dedicated section hereafter.

M-MDSCs and conventional monocytes share a similar phenotype, with however as main differences, lower expression of MHC Class II molecules and immunosuppressive capability for M-MDSC (53). However, “classical” CD14^hi^CD16^lo^ monocytes may also exhibit low expression of HLA-DR, which is further reduced in the context of inflammation, sepsis, or cancer. In fact, cells with such a monocyte/M-MDSCs phenotype in the context of cancer have been shown to be immunosuppressive, blocking antitumoral T cell responses (54). In addition, both cell types have been reported to differentiate into pro-tumorigenic TAMs. This suggests a change of function of monocytes induced in the context of cancer (or other pathological conditions), rather than the occurrence of two different cell subtypes. Therefore, immunosuppressive monocytes and M-MDSC substantially overlap phenotypically and functionally. It is also noteworthy that some cells of myeloid origin exhibiting phenotypic characteristics that do not meet the classical definition of MDSC because they lack expression of specific MDSC markers (but which are nonetheless immunosuppressive and pro-tumoral) are excluded by this current MDSC terminology and may thus be overlooked. This is for instance the case of non-classical monocytes described in different cancers (55–58).

Overall, the “MDSCs” terminology is a conceptual approach, which was indispensable fifteen years ago to provide a comprehensive picture of a particular phenomenon observed in many cancers: the expansion of a myeloid cell population, more or less mature, with immunosuppressive properties. With the evolution of detection technologies such as scRNAseq, new knowledge of these cells has been brought, and it appears today as an essential requirement to regroup these myeloid cells according to their refined phenotype, in order to better identify and ultimately target them. However, since many studies do not allow such a discrimination (because of the use of an incomplete phenotype to identify these cells), in the next section, we will discuss the “all” MDSCs population (CD11b^+^/Gr1^+^ in mice, CD33^+^ in humans), the “early MDSCs” and the monocytic fraction (monocytes and M-MDSCs).

### “MDSCs” in Breast Cancer Patients

In breast cancer patients, while some studies have reported that immunosuppressive monocytic cell number is increased compared to control patients (54), others did not observe this expansion (59). These cells have been associated with more advanced disease (60), and with a worse survival (61). Increased eMDSC numbers have also been correlated with a worse response to neo-adjuvant chemotherapy in TNBC patients (62). In breast cancer tissues, many reports have described, with various degree of accuracy, myeloid cells exhibiting an immature phenotype (CD33^+^CD13^+^CD14^-^CD15^-^) and immunosuppressive properties, which have been associated with adverse prognostic features (higher tumor grade, positive lymph nodes) (63). The composition of this myeloid infiltrate was different among studies, composed either with a majority of CD14^+^ monocytic immunosuppressive cells (64), or with granulocytic myeloid cells and early MDSCs (59). Together, these studies consistently advocate for the presence and role of suppressive myeloid cells in the tumor microenvironment, but their contradictory findings related to the exact phenotype of these cells highlights the extreme heterogeneity of this myeloid landscape, as further outlined more recently in scRNAseq and cytometry by time of flight (CyTOF) studies discussed in the next sections (15, 16).

### Direct Effects of “MDSCs” on Tumor Cells

In primary tumor sites, suppressive myeloid cells recruited by cancer cells play an important role in inhibiting anti-tumor immune responses using many mechanisms extensively reviewed elsewhere (expression of ARG1, production of NO, ROS, and prostaglandin E2) (50, 51). However, many studies have also lent support to the notion that, besides their role as potent suppressor of cancer immunity, “MDSC” may also play an important role in breast cancer cell invasion, activate other stromal cells such as fibroblasts, and promote angiogenesis. Recruited at the hypoxic tumor sites, CD11b^+^Gr1^+^ MDSCs produce S100A8, an alarmin not only involved in the recruitment of additional MDSCs, but also implicated in the activation of endothelial cells. This activation led to the modification of tight junctions, leading to vascular leakage (65).

MDSCs also participate to breast cancer resistance to chemotherapy. It has indeed been shown that immunosuppressive CD33^+^ cells isolated from breast cancers patients are able to induce a stemness phenotype (associated with cancer cell chemoresistance) in the breast cancer cell line MCF-7 (64). Furthermore, the chemotherapeutic agent doxorubicin has been found to increase the levels of monocyte chemoattractant proteins (MCPs) 1 to 3 and particularly MCP1/CCL2 (48, 66). This chemotherapy-induced expression of CCL2 has also been reported in the metastatic sites such as the lungs. It results from the release of extracellular vesicles enriched in annexin-6 by chemoresistant cancer cells, which induced the recruitment of Ly6C^+^CCR2^+^ monocytes that participate to the pre-metastatic niche formation (48).

### Preparation of the Metastatic Niche by Recruited “MDSCs”

CD11b^+^Gr1^+^ granulocytic and monocytic myeloid cells critically contribute to the metastatic dissemination of breast cancer cells (52). It has been observed that in the mouse 4T1.2 mammary cancer model, pro-inflammatory monocytes with MDSCs features (IL4R, CD49b, CD62L, CD11b) can be recruited by the chemokine CCL2 in the pre-metastatic lungs where they foster metastasis. CCL2 promotes the release of the alarmin S100A8/9 which further increases MDSC recruitment (67). CD11b^+^Gr1^+^ cells have been reported to progressively accumulate in the lungs of 4T1 tumor-bearing mice before the arrival of cancer cells, and to prepare the lung environment for seeding by metastatic cells *via* vascular remodeling and production of MMP9 (68). Interestingly, this recruitment of CCR2^+^ cells induced by inflammatory signals can be mediated by other sources of inflammation than tumors. Indeed, myocardial infarction represent a major cause of systemic stress and is accompanied by systemic monocytosis. It has been shown to be associated with a higher risk of relapse and cancer-specific mortality in early breast cancer patients (69). In tumor-bearing mice, myocardial infarction results in an important recruitment of Ly6C^+^ monocytes with immunosuppressive functions, which can differentiate into pro-tumorigenic macrophages at the tumor sites and accelerate primary tumor growth and metastasis (69). It has also been reported in the 4T1 triple negative mouse breast cancer model that Gr1^+^ cells primarily promote the metastatic cascade by facilitating extravasation of malignant cells at the distant metastatic lungs through IL1β and matrix metalloproteinase secretion (70). Along these lines, CXCR4-dependent mechanisms were involved in Gr1^+^ cell-mediated metastasis promotion in a mouse breast cancer model (71)

At the future metastatic site, MDSCs are implicated in the angiogenic switch. In two different studies in 4T1-bearing mice, CD11b^+^Gr1^+^ cells recruited in the lungs have been demonstrated to upregulate many pro-angiogenic factors such as *Il1ß, Mmp9, Tnf, Tie2* (72), or to secrete platelet-derived growth factor-BB (PDGF-BB), which mediates angiogenesis (73).

### Differentiation of “MDSCs” Into Other Cell Types in the Context of Breast Cancer

“MDSC” are endowed with a particularly high degree of plasticity. Indeed, many reports indicate that monocytes/M-MDSCs often differentiate into pro-tumorigenic macrophages at the tumor site or in the metastatic organs. This phenomenon has been tracked *in vivo* with the use of GFP^+^ expressing myeloid cells, transferred into E0771-bearing C57BL/6 mice: few hours after the transfer, classical monocytes were recruited in the metastatic lungs, where they differentiated into macrophages precursors, before becoming metastasis-associated macrophages (74). Exosomes derived from mesenchymal stem cells can promote the differentiation of M-MDSCs into highly immunosuppressive pro-tumorigenic macrophages (75).

Bone is one of the most important metastatic sites in breast cancer patients, with up to 70% of metastatic patients facing bone metastasis. MDSCs and monocytic cells play a major role in the formation of this metastatic site. Indeed, it has been demonstrated that MDSCs (defined as CD11b^+^Gr1^+^) cells can differentiate into osteoclasts *in vitro* and *in vivo*. These osteoclasts are capable of bone resorption (76, 77).

## Immunosuppressive Myeloid Cells of the Granulocytic Lineage: Versatile Tumor-Promoting Functions in Breast Cancers

### Granulocytic Cells in the Breast TME: Phenotypes, Functions and Controversies

Neutrophils constitute the more prominent leucocytes, primarily participating to the first lines of defense against infectious agents. They are produced in the bone marrow from granulocyte-monocyte myeloid progenitors (GMPs), which originate from lymphoid-primed multipotent progenitors, themselves derived from hematopoietic stem cells. Their maturation and differentiation depend on G-CSF and STAT3 activation.

In the context of cancer, and particularly in the context of breast cancers, it has been demonstrated that malignant cells could disrupt neutrophil homeostasis, hijacking their production and functions to their advantage through the production of TDFs such as G-CSF (78). The phenotypic characterization of tumor-associated neutrophils and the identification of specific subsets have remained the matter of intensive debates for the past few years. As mentioned in the previous section, the discrimination and possible relationship between tumor-associated neutrophils and polymorphonuclear (PMN)-MDSCs (MDSCs with a granulocyte phenotype) has remained an outstanding question in the field. Whether PMN-MDSC and tumor-associated neutrophils represent the same cell populations or are different subsets remains highly questionable. Indeed, in human, both are commonly identified as SSC^high^, CD33^+/medium^, CD11b^high^, CD16^+^, CD15^+^, CD66b^+^, HLA-DR^neg^. Furthermore, many preclinical studies, primarily in mouse cancer models, on which most of our understandings of tumor-associated neutrophils have been based, do not clearly distinguish between neutrophils and PMN-MDSC. In these studies, cells with a CD11b^+^Gr1^+^Ly6G^+^LY6C^med/low^ phenotype have been shown to expand over the course of cancer progression, and functional assays to assess their immunosuppressive properties have not always been performed (79, 80).. Although some markers have been proposed to discriminate between PMN-MDSC and “classical neutrophils” such as LOX-1 (81), CD84 and JAML (82), or sometimes the alarmins S100A8 or S100A9 (50), PMN-MDSC have mainly been defined by their immunosuppressive properties. Along these lines, because neutrophil density is higher than that of MDSC, it has also been proposed that density gradients may be used to separate physically tumor-associated neutrophils and tumor-induced PMN-MDSC. However, mature neutrophils have also been shown to exhibit immunosuppressive and pro-tumorigenic features in the TIME, and activated neutrophils fall in the “low-density” section of density gradients. Some authors also tried to establish a “N1/N2” dichotomy similar to that proposed for macrophages, N2 being pro-tumorigenic, immunosuppressive neutrophils (83). However, to date no reliable marker allow for a clear distinction of neutrophil different differentiation stages, and neutrophils in cancer are likely present as a heterogenous population, with cells at various activation states (84).

Recent reports have attempted to address this equivocal identity of PMN-MDSC and tumor-associated neutrophils. A single-cell transcriptomic analysis of the myeloid compartment in the splenocytes from two tumor-bearing mice (PyMT tumor model) and 2 tumor-free control animals has suggested that PMN-MDSCs differ from their normal myeloid counterparts and may originate from neutrophil progenitor cells undergoing an aberrant differentiation path (82). Data from another recent study have suggested that peripheral PMN-MDSCs from patients with metastatic breast cancer are more closely related to healthy donors’ neutrophils than to MDSCs induced in another pathological condition (Gram-positive sepsis) (85). Further mass-cytometry analysis revealed that unique subpopulations of these granulocytic cells were specifically present in cancer patients, with a majority of low density mature activated neutrophils and a minority of immature neutrophils lacking maturation markers (CD10, CD13, CD45) at different maturation stages. These cells were collectively referred to by the authors as “G-MDSCs” and proposed to constitute neutrophils at various differentiation stages (85). These data thus advocate for a differential differentiation and activation profile of neutrophils in the context of cancer.

**Figure 2:**
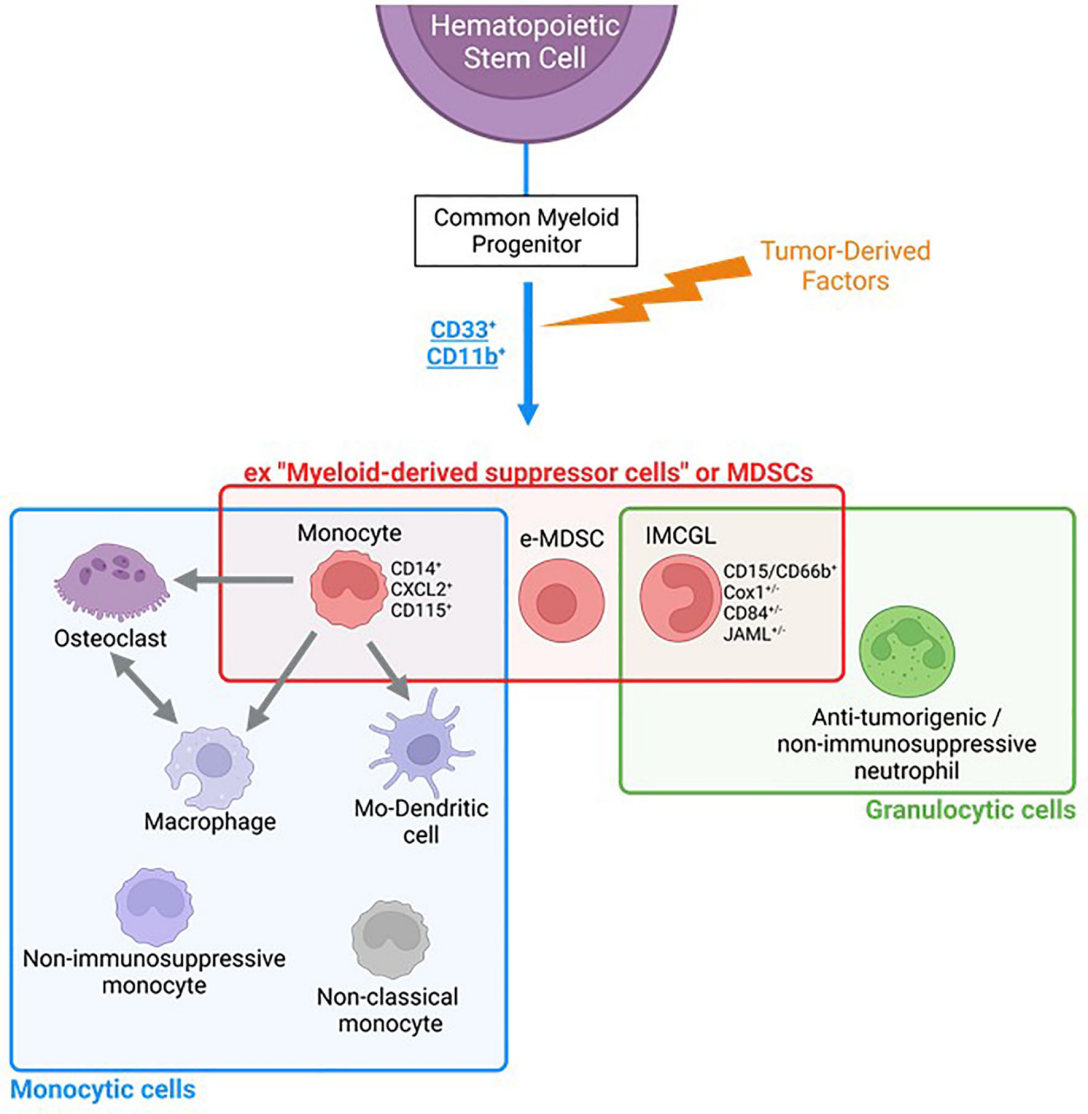
Overlaps between subsets of tumor-promoting myeloid cells (monocytic vs gralulocytic origine). Cells formerly referred to as “Myeloid-Derived Suppressor Cells, MDSCs” encompass undifferentiated CD33^+^CD11b^+^ (“early-MDSCs”), immunosuppressive cells of the granulocytic lineage that we propose to call “immunosuppressive Myeloid Cell of the Granulocytic lineage, IMCGL” (“formal PMN-MDSCs”), and monocytes endowed with pro-tumoral properties. These tumor-promoting monocytes (“formal m-MDSCs”) can differentiate into pro-tumoral macrophages, Mo-dendritic cells or osteoclasts, each endowed with multiple dedicated tumor-promoting activities. IMCG, immunosuppressive myeloid cells from granulocytic lineage; e-MDSCs, early-myeloid-derived suppressor cells; Mo-Dendritic cell, monocyte-derived dendritic cells.

Considering this significant phenotypic and functional overlap between so-called “PMN-MDSC” and “immunosuppressive neutrophils” we propose to refer to these cells more accurately using the term “immunosuppressive myeloid cells of the granulocytic lineage” or “IMCGL” until definitive phenotypic markers or functional assays are available to unequivocally distinguish them. (**Figure 2**). We believe that, compared to the terms “immunosuppressive neutrophils” or “PMN-MDSC”, the denomination ““IMCGL” allows to group these highly overlapping cell types to better study them and partly address the current controversies and challenges in distinguishing between immunosuppressive neutrophils and PMN-MDSC (**Table 1**). The physiological relevance and clinical usefulness of discriminating these cells also remains to be addressed.

**Table 1:** IMCGL terminology, phenotype and non-immunological functions.

*Study*	*BC subtype*	*Model*	*Cell line*	*IMCGL name in original article*	*Phenotype*	*Functions*	*Additional findings in patients*
*Ouzounova et al., Nat Commun 2017* (52)	TNBC	Mice	4T1	MDSCs	CD11b^+^ Ly6G^+^ Ly6C^lo^	MDSCs promote primary and disseminated tumor cell growth and promote the formation of lungs metastasis.	
*Kim et al., Nat Cell Biology 2019* (86)	TNBC	Mice	2208L, PyMT-N, 4T1, AT3, PyMT-M, E0771, 67NR	TINs (encompass both neutrophils and MDSCs)	CD45^+^, CD11b^+^, Ly6G^+,^Ly6C^med-low^	Creation of an immunosuppressive environment which impairs response to ICI	
*Wang et al. CCR 2019* (90)	HR+, TNBC, HR+/HER2+	Human (*in vitro*)	MCF-7, MB-231, BT-474	Neutrophils isolated from patients tumors	CD11b^+^ CD66b^+^	Promotion of migration and invasion, + induction of EMT (tried on MCF7 cells only)	Prognosis: stromal CD66b+ cells (IHC) are associated with poor prognosis, advanced histologic grade, tumor size, LN metastasis, high TNM stage, TNBC and distant metastasis
	TNBC	Mice	4T1	Neutrophils	Ly6G^+^	Promotion of metastasis. Use of an anti Ly6G+ antibody prevented the formation of lungs and liver metastasis.	
*Liang et al, PNAS 2018* (92)	TNBC	Mice	4T1	Neutrophils	Ly6G^+^	Promotion of tumor growth and metastasis (via transferrin secretion)	Peripheral neutrophils from breast cancer patients with metastatic disease, isolated by an immunomagnetic negative selection, promoted tumor cell growth of MDA-MB-231 cells *in vitro*.
*Xiao et al, Cancer Cell 2021* (93)	TNBC	Mice	4T1, 4T07	Neutrophils	CD11b^+^ Ly6G^+^	Via the formation of NETs, neturophils recruited at the metastatic niche promote cancer cell proliferation	NETs and neutrophil infiltration are higher in lung metastases than in primary tumors (neutrophils: MPO in IHC). This infiltration is higher in TNBC than in HR+ tumors.
*Kumar et al., JCI 2018* (96)	TNBC	*In vitro*	HCC1806, SUM159	MDSCs	CD45^+^, CD11b^+^, Ly6G^+^/Ly6C^lo^	Addition of MDSCs in turmospheres assay significantly increased the number of tumorspheres	MDSCs infiltration is higher in TNBC cancers (CD11b^+^ CD33^+^ S100A9^+^ CD15^+^ LOX1^+^)
*Luo et al., J Breast Cancer 2020* (97)	HR+, TNBC	*In vitro*	MCF-7, MDA-MB-231	MDSCs	CD33^+^HLA-DR^-^CD15^+^ in PBMCs from BC pts	MDSCs promote migration and invasion of BCC *via* induction of EMT	The frequency of MDSCs increases in peripheral blood of patients with breast cancer. This was higher in high stage patients (III/IV), with extensive tumor burden, lymph node metastases, distant metastases, poorly differentiated tumors.
*Vazquez Rodriguez et al., Cancer Immunol Res 2017* (102)	HR+	Zebrafish	MCF-7	Neutrophils	LFA-1 (neutrophils from HD, obtained by Ficoll)	Neutrophils promoted migration and invasion at tumor site, and intra/extravasation. They comigrated with cancer cells	
*Szczerba et al., Nature 2019* (103)	HR+, TNBC, HER2+	Mice (immunocompetent and nude)	BR16, LM2, 4T1, PyMT	Neutrophils	Ly6G^+^	Neutrophils directly interact with CTCs to support cell cycle progression in circulation and to accelerate metastasis seeding	Clusters of neutrophils and circulating tumor cells were also found in breast cancer patients blood samples. These clusters were associated with a significantly worse prognosis.
*Sprouse et al., Int J Mol Sci 2019* (104)	HR+, TNBC, HER2+	Human	Isolated from patients	MDSCs	CD45^+^/CD33^+^/CD11b^+^/CD14^-^/CD15^+^	MDSCs from patients formed clusters with CTCs, as seen in patients and reproduced *in vitro*. This companionship promoted migration and proliferation of cancer cells.	High number of MDSCs is correlated with metastasis in BC patients. PMN-MDSCs are responsible for higher ROS levels in the plasma of these patients.
*Hsu et al., Cell Rep 2019* (106)	TNBC	Mice	4T1	“low-density” neutrophils	Low fraction of Ficoll, CD11b^+^Ly6G^+^	Promotion of liver metastasis formation, *via* NETosis	
*Sangaletti et al., Front Immunol 2019* (107)	HER2+	Mice	SN25A, N3D + co-isogenic transfected with LXSPARCSH	MDSCs	CD45^+^, CD11b^+^, Ly6G^+^	MDSCs promote tumor growth, angiogenesis and EMT in cancer cells.	
*Albrengues et al., Science 2018* (110)	HR+	Mice (immunocompetent and nude)	D2.0R, MCF-7	Neutrophils	Ly6G^+^ and/or MPO and/or Ficoll density gradient	Neutrophils promoted awakening of dormant cancer cells	
*Park et al., Sci Transl Med 2016* (111)	TNBC	Mice	4T1, tumors from C3(1)-Tag mice	Neutrophils	Ficoll + CD45^+^, Ly6G^+^, CD11b^+^	Neutrophils are recruited by cancer cells at the metastatic sites where they promote tumor cells growth, migration and invasion *via* the formation of NETs.	NETs are detectable in human breast cancers and are more frequent in more agressive cancers (TNBC vs HR+).
*Yang et al., Nature 2020* (112)	TNBC	Mice	4T1, MDA-MB-231	Neutrophils	MPO (IHC)	NETs at metastatic site promote migration and adhesion of cancer cells, and the formation of metastasis.	NETs are present in metastatic lesions of breast cancers such as liver, lungs, bones and brain, liver metastases having the most abundant infiltration (IHC: MPO and H3Cit stainings). Serum NETs level (detection of MPO/DNA complex) are significantly higher in patients with liver metastases. High levels of MPO/DNA was an independent variable associated with metastasis to the liver.
*Teijeira et al, Immunity 2020* (113)	TNBC	Mice	4T1	Both	Ly6G^+^	NETs formed by neutrophils and/or MDSCs shield tumor cells from NK cells and help them avoid immune destruction, thus leading to the formation of more metastasis.	
*Ouzounova et al., Nat Commun 2017* (52)	TNBC	Mice	4T1	MDSCs	CD11b^+^ Ly6G^+^ Ly6C^lo^	MDSCs promote primary and disseminated tumor cell growth and promote the formation of lungs metastasis.	

BC, breast cancers; EMT, epithelial-to-mesenchymal transition; ICI, immune checkpoint inhibitors; HR+, hormone receptor positive; IHC, immunohistochemistry; IMCGL, immunosuppressive myeloid cells of granulocytic lineage; LN, lymph node; MDSCs, myeloid-derived suppressor cells; MPO, myeloperoxydase; NETs, neutrophils extracellular traps; PBMCs, peripheral blood mononuclear cells; TINs, tumor-infiltrating neutrophils; TNBC, triple-negative breast cancer; TNM.

### IMCGL in Breast Cancer Patients

In breast cancers, IMCGL have historically been considered as a major obstacle to anti-cancer immunity because of their immunosuppressive activities, a concept supported by recent findings in triple-negative breast cancer mouse models, where IMCGL-infiltrated tumors do not respond to immunotherapy (86). In breast cancer patients, tumor-induced expansion of circulating IMCGL has been corelated with a worst prognosis (87, 88). Interestingly, IMCGL are more frequently found in the tumor beds than in “healthy” adjacent tissues (59), and in most studies IMCGL have been associated with a higher tumor stage (89), or with a worse prognosis and an impaired response to chemotherapy (90). Tumor-induced IMCGL have been reported to impair T cell activation, particularly in advanced tumors, through increased production of ROS, NO or ARG1 or expression of immunoinhibitory ligands such as PDL1 (91).

### IMCGL at the Primary Tumor Sites

IMCGL expansion and recruitment is directly promoted by breast cancer cells by various mechanisms. GM-CSF secretion by malignant cells induces the production of transferrin in Ly6G^+^ cells from 4T1-bearing mice that, in turn significantly enhances primary tumor growth *in vivo* and *in vitro*. In humans, the transferrin, *TFR1* gene has been found to be up-regulated in breast cancers, and higher levels of this protein have been associated with higher tumor grades/stages, but also with a significantly worse survival (92). It has been shown that G-CSF production by breast cancer cells induces the recruitment of IMCGL that accumulate in the periphery of tumor-bearing PyMT mice (78). Cathepsin C (CTSC) produced by cancer cells has also been reported to induce recruitment and activation of IMCGL in 4T1 tumor bearing-mice. CTSC expression in human breast cancer is associated with metastasis and IMCGL occurrence (93). IMCGL recruitment can also be indirectly promoted by IL-1ß-secreting TAMs, which are recruited by Wnt ligands in p53-deficient cancer cells (94), or by cancer cell secretion of CCL2 (37).

Besides being equipped with immunosuppressive properties, IMCGL are also endowed with versatile tumor-promoting functions. Indeed, IMCGL can also induce and promote angiogenesis, participate to the remodeling of the extracellular matrix, contribute to tumor cell invasion, and participate to metastatic dissemination (84). IMCGL have also been shown to form Neutrophil Extracellular Traps (NETs) involved in tumor cell capture and growth as detailed hereafter (95).

IMCGL have been reported to display direct effects on breast cancer cells. Recent studies have indeed demonstrated that these cells participate to the acquisition of a stem-cell phenotype by malignant cells. Breast cancer cells expressing the ΔNp63 protein secrete CCL22 and CXCL2 that recruit IMCGL, which, in tumorsphere assays, promote the stemness phenotype of breast tumor cells *via* the secretion of CHI3L1 and MMP9 (96). Along these lines, it has also been reported that tumor-infiltrating IMCGL from breast cancer patients induce EMT in the MCF-7 cancer cell line, and promoted migration and invasion in *in vitro* assays (90). Consistent with these data, CCL3-recruited IMCGL have been observed to foster the EMT in breast cancer cells and enhance their proliferation, migratory and invasive properties through the PI3K/Akt/mTOR pathway (97).

### IMCGL in the Metastatic Process

Most studies in the field have however shown that, primarily, IMCGL foster the process of breast cancer invasion and metastasis, with limited effects on primary tumor growth. Indeed, the elimination of these cells in pre-clinical models resulted mainly in dampening metastatic dissemination, with limited influence on primary tumor development (90, 94, 98–101). In zebrafish injected with MCF-7 cells, IMCGL from healthy human donors are capable of promoting cancer cell migration and intravasation at the tumor injection site in an Estradiol-dependent manner. In this model, IMCGL have been shown to migrate together with circulating cancer cells and to extravasate together in distant sites, thereby supporting disseminated tumor cell establishment in new metastatic niches (102). Two recent reports have brought substantial new insights into this joint migration of IMCGL with cancer cells. Indeed, evidence has emerged that some subpopulations of IMCGL can form companionship clusters with circulating tumor cells (CTC), and chaperone these CTC, protecting them in the circulating blood. Furthermore, IMCGL may foster CTC seeding at distant sites. CTC primarily circulate alone in the peripheral blood where the vast majority die in this environment. Only a limited number of these CTC (2-4%) have been detected as circulating homotypic or heterotypic clusters (103). These clusters have been associated with a significantly worse prognosis in breast cancer patients (103, 104). Heterotypic clusters are composed of CTC with white blood cells, most of them being myeloid cells of the granulocytic lineage (103, 104). Sprouse et al. have recently explored the cross-talks in these clusters between CTC and CD33^+^CD11b^+^CD15^+^ IMCGL cells (defined by the authors as PMC-MDSC) in the PBMC fraction of the peripheral blood (104). IMCGL induce upregulation of Notch1 receptor expression in CTCs through the ROS-NRF2-ARE axis, while CTCs induce pro-tumorigenic differentiation of IMCGL through paracrine Nodal signaling. Importantly, in mice, co-injection of breast cancer cells with IMCGL leads to an early dissemination of malignant cells to the lungs and brain (104). Szczerba et al. have also studied the interactions between CTC and IMCGL in heterotypic clusters from breast-cancer patients and from mammary tumor-bearing mice (103). Within these clusters, CTC exhibit a marked enrichment in positive regulators of cell cycle and DNA replication programs. Furthermore, RNA sequencing analysis has identified cytokines implicated in these cellular cross-talks and determined that IMCGL secrete TNFα, OSM, IL-1ß and IL-6, while CTC express CSF1, CSF3 (G-CSF), TGF-ß and IL-15 (103). However, the exact identity of each cellular partners within these clusters, the nature and importance of their interactions as it relates to the metastatic process, and the mechanisms underlying the promotion of CTC seeding and development at the metastatic niches remain to be fully uncovered. Along these lines, not only do IMCGL represent the main immune cells present at the metastatic site (105), but they also critically participate to the preparation of pre-metastatic niches. In tumor-bearing mice, myeloid cells accumulating in distant tissues are essentially composed of Ly6G^+^ immunosuppressive IMCGL (78). In 4T1 tumor-bearing mice, accumulation of IMCGL in the lungs (52) or in the liver (106) promotes metastatic cancer growth, and disseminated malignant cell proliferation. Importantly, depletion of IMCGL with an anti-Ly6G antibody suppresses metastasis in both studies (52, 106). It is noteworthy that although Ly6G has been used to deplete granulocytic cells, whether this approach results in the elimination of all IMCGL subsets remains to be determined.

### Importance of NETs in Breast Cancer

Neutrophils have been described for their capacity of releasing neutrophil extra-cellular traps or “NETs”, a function that has also been described for PMN-MDSCs (107). NETosis is the process by which neutrophils release large web-like structures composed of cytosolic and granule proteins assembled on de-condensed chromatin. NETosis has been proposed to be a specific defense mechanism harbored by neutrophils against some pathogens like funga. The phenomenon of NETosis has also been observed in cancer where it can be triggered in part by G-CSF produced by many malignant cells (95). The impact of this process on cancer progression and on disease-associated complications such as thrombosis is being increasingly acknowledged (95, 108). A recent breakthrough in breast cancer was the findings that NETs may contribute to the awakening of dormant cancer cells. Reactivation of dormant cancer cells is of utmost importance in breast cancers, since half of patient relapses occur more than 5 years after the initial diagnosis, and in some cases even up to 20 years (109). Using breast cancer models that usually do not metastasize in mice, Albrengues et al. have demonstrated that neutrophil-derived NETs, induced by inflammatory conditions such as prolonged tobacco exposure or LPS instillations, lead to dormant cancer cell awakening and development into aggressive lungs metastases. In this setting, inflammation triggers NETs extrusion, which forms a scaffold allowing the sequential cleavage of laminin by neutrophil elastase (NE) and MMP9, as well as thrombospondin 1 (TSP-1). This laminin cleavage activates an α3β1-associated signaling in dormant cancer cells, leading to their reactivation (110). TSP-1 is a key matricellular protein that has been reported to inhibit metastasis. As outlined hereabove, CTSC secreted by cancer cells promotes the recruitment and activation of neutrophils in the metastatic niches, which upon activation form NETs that degrade the extracellular matrix, in part by cleavage of TSP-1, thereby allowing cancer cell proliferation and establishment (93). Other reports have indicated that in the context of breast cancers, NETs actively contribute to the formation of the pre-metastatic niches (111, 112). Indeed, these structures have been observed in the lungs of mice early after injection of 4T1 cells, thus before arrival and seeding of breast cancer cells. Furthermore, evidence has been provided that, *in vitro*, NETs stimulate invasion and migration of cancer cells. Consistently, NETs digestion with deoxyribonuclease I (DNase I) has been reported to significatively reduce the occurrence of lung metastasis (111). In humans, NETs have been detected in large amount in the metastatic lungs, and circulating NETs levels are higher in metastatic breast cancer patients compared to early-stage cancer patients (93). Suggesting a role of NETs in metastatic tumor cell organotropism regulation, Yang et al. have demonstrated that NETs contribute to the formation of metastases in the liver but not in the lungs. Furthermore, the authors have identified the protein CCDC25 expressed at the surface of cancer cells as a specific sensor of NETs DNA, and responsible for malignant cells migration, adhesion and proliferation induced by NETs. In breast cancer patients, CCDC25 has been detected in cancer cells with a clear membrane staining at the border of the tumor, and higher levels of CCDC25 in the primary tumors have correlated with a reduced survival (112).

Finally, NETs act as “shields” for cancer cells, wrapping them to avoid destruction by cytotoxic CD8 T cells of NK cells, adding to their multiple tumor-promoting functions (113).

## Roles of “Tolerogenic/Regulatory” Dendritic Cells in Breast Cancer Development

### Dendritic Cell Alterations in Breast Cancers

Dendritic cells (DC) play a central role in cancer immunosurveillance. They capture antigenic material from neoplastic cells, process tumor-specific antigens and present the derived peptides onto MHC class I or class II. Upon migration to the secondary lymphoid tissues, they activate effector tumor-specific CD8^+^ CTL and CD4^+^ Th lymphocytes. DC can also promote the anti-tumoral functions of NK, NKT and γδ T cells (114). However, in most cancers, DC are phenotypically and functionally impaired leading to dampened anti-cancer immunity (115). Although the nature of the microenvironment of breast cancers greatly varies depending on the tumor subtype and stage of the disease, in most cases it negatively influences DC capability to induce and sustain anti-tumor immunity. These DC alterations in breast malignancies have been attributed: a) to DC elimination; b) to the blockade of the generation of these cells from DC precursors; c) to the triggering of functional deficiency in DC (reduced antigen capture, processing, presentation and ability to activate T lymphocytes); and d) to the generation of immunosuppressive and tolerogenic DC capable of blocking anti-cancer T cells, inducing T lymphocyte anergy or inducing tumor-promoting regulatory T cells (Treg) (116–118). These defects are induced by different tumor-derived factors [extensively reviewed in (116, 117)], among which are VEGF (119), TGFβ (120), IL10 (121), PGE2 (122) or tumor-produced polyamines (123), and are responsible for a deficient induction of anti-cancer T lymphocyte proliferation and activation, thus contributing to breast cancer evasion from immunosurveillance.

In addition, different studies have demonstrated that breast malignancies are associated with the induction of different subpopulations of DC (myeloid, mDC or plasmacytoid, pDC) at different stages of maturation in the tumor, lymph nodes or blood, which actively promote T cell anergy and suppression and/or which trigger tumor-promoting Treg induction by a variety of mechanisms such as L-Arginine depletion (124), PD-L1 (125), TGFβ, IDO (126) or ICOS-ligand (127). Breast cancer-derived thymic stromal lymphopoietin (TSLP) has been identified as an inducer of OX40L on DC infiltrating primary breast cancer (128). These OX40L^+^ DC participate to the induction of IL-13- and TNF- producing Th2 cells thus contributing to the promotion of an environment permissible for breast tumor growth (128).

### Role of DC in Breast Cancer Angiogenesis and Metastasis

Although many studies have extensively reported on the immune-modulatory role of tolerogenic/regulatory DC in breast cancers, much sparser reports are available as it relates to the tumor-promoting pro-angiogenic, pro-invasive and pro-metastatic properties of these cells. In this context, a study has correlated the presence of immature DC in highly angiogenic tumors (129), but the mechanistic bases underlying neoangiogenesis promotion remains to be determined. Similarly, the role of DC in breast cancer metastasis remains incompletely elucidated. A recent report indicates that CD303^+^ pDC accumulating in human breast cancer beds of patients with positive lymph nodes promote CXCR4 expression by cancer cells, suggesting that these tumor-associated pDC may participate to malignant cell metastasis to lymph nodes expressing SDF-1 through a CXCR4/SDF-1-dependent mechanism (130). Since DC conditioned by the tumor microenvironments can produce TGFβ, these cells may also contribute to the epithelial-mesenchymal transition (EMT) precluding tumor cell migration from primary tissues to metastatic sites, but a formal demonstration of this effect in breast cancer remains to be provided. Likewise, the possibility that DC may contribute to the preparation of pre-metastatic niches, before seeding of metastasizing cancer cells has yet to be formally demonstrated. In this context, a recent study has suggested that, in the mouse breast cancer model E0771, glucose-regulated protein 78 (GRP78) produced by tumor reduces DC MHC class II expression in the liver in the early stage of metastasis. However, the actual role of DC in the preparation of the pre-metastatic liver has not been demonstrated (131). Along these lines, recent data have also indicated that CD11c^+^ DC exposed to conditioned medium of RANKL^+^ T cells from the bone marrow of 4T1 mammary tumor-bearing mice can differentiate into osteoclast-like cells, suggesting that DC may participate to the osteolytic process occurring in metastatic breast cancer patients (132).

## Conclusion, Perspectives and Challenges

The critical contribution of immune cells of the myeloid lineage to the mechanisms of cancer escape from immune detection and elimination is now widely recognized, and many studies have deciphered the various modes of action underlying the immunosuppressive properties of these cells. The notion that, beside this cardinal role in antitumor immunity, different myeloid cell populations are also endowed with a variety of “non-immunologic” tumor-promoting functions has drawn less scrutiny, until recently.

The heterogeneous nature of tumor-promoting myeloid cells, with some likely phenotypical and functional overlaps between subsets (illustrated in **Figure 2**) remains a major challenge preventing the unequivocal identification of distinct subpopulations. This current problem is probably best illustrated by the difficulty to draw a clear line between PMN-MDSC and immunosuppressive neutrophils in breast cancer patients or in mammary tumor models. Recent extensive studies, which have attempted to establish dedicated genomic, proteomic and biochemical profiles to better characterize these cells, have actually highlighted further the complexity and the high degree of plasticity of this myeloid landscape, advocating for instance that PMN-MDSC may actually correspond to neutrophils at different maturation stages in breast malignancies. These considerations have prompted us to refer to these tumor-promoting granulocytic cells occurring in cancer as “immunosuppressive myeloid cells of the granulocytic lineage, IMCGL”, which, we believe, better depicts their origins and functions. It would be clinically relevant to clearly decipher whether IMCGL are constituted of different subsets with dedicated properties and predictive or prognostic values. Better identification of these cells is a prerequisite to further determining whether they may serve as useful biomarkers and therapeutic targets, which warrants the urgent need to discover novel marker(s) and/or strategies allowing for a clear discrimination of the multiple subsets of these myeloid cells.

An additional outstanding question that still needs to be fully addressed relates to the “division of labor” among these tumor-promoting myeloid cell populations. As outlined in the previous section, it appears that all the main myeloid populations, TAM, MDSCs, IMCGL, and to some extend DC, are endowed with the capacity to exert many pro-tumoral activities. Recent single cell transcriptomic analysis suggest that, within each of these populations, a dedicated subset or even a single cell, may be equipped with concomitant multitasking activities (through the co-expression of factors involved for instance in immunosuppression, extracellular matrix remodeling, metastasis promotion…). The possibility that dedicated subsets or individual cells may sequentially acquire and lose one of these properties at a given time and depending on the nature of their environment and therefore on their location and on tumor stage is also conceivable, but remains to be clearly demonstrated. This functional plasticity of tumor-associated myeloid cells over time and space may be essential to fulfill the specific needs of growing tumors at each of the sequential stages of their development in the primary tumor sites (promotion of tumor growth, EMT, invasion, angiogenesis, intravasation, immunosuppression, production of chemokines involved in the recruitment of tumor-promoting cells), as CTC in the bloodstream (shielding in heterophilic clusters), and in the pre-metastatic and metastatic niches (soil preparation, ECM remodeling, extravasation, chemoattraction, immunosuppression).

Lastly, questions remain concerning the differences between breast cancer subtypes. Few studies have studied precisely the myeloid landscape and compared the different subtypes. Recent RNAseq findings show that myeloid infiltration is present in all main subtypes (“luminal” or HR positive BC, HER2+, TNBC) at different levels (133). A majority of studies discussed in this review focus on the TNBC subtype, some showing a higher infiltration of IMCGL in the tumors of these patients (90, 93, 96, 111). Macrophages are very represented across the different subtypes (133), though their role and exact phenotype in each subtype is unclear.

As these myeloid cells are essential contributors to many tumor-promoting networks, their therapeutic targeting (elimination, inactivation, reprogramming) has logically led to promising anti-tumor responses. However, the high phenotypic and functional heterogeneity and plasticity of these cells over time and depending on their tissue location has, to date, been a major hurdle for both their use as definitive biomarkers and the development of therapeutic strategies that would specifically interfere with their generation, development and multifaced tumor-promoting functions, which underlines the need to further characterize this myeloid landscape.

## Author Contributions

CB and NL conceived and wrote the article. TB drew figures and wrote the article. FP and CD wrote the article. All authors contributed to the article and approved the submitted version.

## Funding

Supported by the French national league against cancer and the SIRIC-BRIO (NL), and the Fondation ARC (CB).

## Conflict of Interest

The authors declare that the research was conducted in the absence of any commercial or financial relationships that could be construed as a potential conflict of interest.

## Publisher’s Note

All claims expressed in this article are solely those of the authors and do not necessarily represent those of their affiliated organizations, or those of the publisher, the editors and the reviewers. Any product that may be evaluated in this article, or claim that may be made by its manufacturer, is not guaranteed or endorsed by the publisher.
